# Hedgehog: A Key Signaling in the Development of the Oligodendrocyte Lineage

**DOI:** 10.3390/jdb4030028

**Published:** 2016-09-08

**Authors:** Elisabeth Traiffort, Mary Zakaria, Yousra Laouarem, Julien Ferent

**Affiliations:** 1 Neuroprotective, Neuroregenerative and Remyelinating Small Molecules’ U1195, INSERM—Université Paris-Sud, Université Paris-Saclay, 80 rue du Général Leclerc, Kremlin-Bicêtre F-94276, France; mary.zakaria@inserm.fr (M.Z.); yousra.laouarem@inserm.fr (Y.L.); 2 IRCM, Molecular Biology of Neural Development, 110 Pine Avenue West, Montreal, QC H2W 1R7, Canada; julien.ferent@gmail.com

**Keywords:** oligodendrocyte, neural tube, forebrain, optic nerve, Smoothened, Patched, Gli

## Abstract

The Hedgehog morphogen aroused an enormous interest since it was characterized as an essential signal for ventral patterning of the spinal cord two decades ago. The pathway is notably implicated in the initial appearance of the progenitors of oligodendrocytes (OPCs), the glial cells of the central nervous system which after maturation are responsible for axon myelination. In accordance with the requirement for Hedgehog signaling in ventral patterning, the earliest identifiable cells in the oligodendrocyte lineage are derived from the ventral ventricular zone of the developing spinal cord and brain. Here, we present the current knowledge about the involvement of Hedgehog signaling in the strict spatial and temporal regulation which characterizes the initiation and progression of the oligodendrocyte lineage. We notably describe the ability of the Hedgehog signaling to tightly orchestrate the appearance of specific combinations of genes in concert with other pathways. We document the molecular mechanisms controlling Hedgehog temporal activity during OPC specification. The contribution of the pathway to aspects of OPC development different from their specification is also highlighted especially in the optic nerve. Finally, we report the data demonstrating that Hedgehog signaling-dependency is not a universal situation for oligodendrocyte generation as evidenced in the dorsal spinal cord in contrast to the dorsal forebrain.

## 1. Introduction

**Figure 1:**
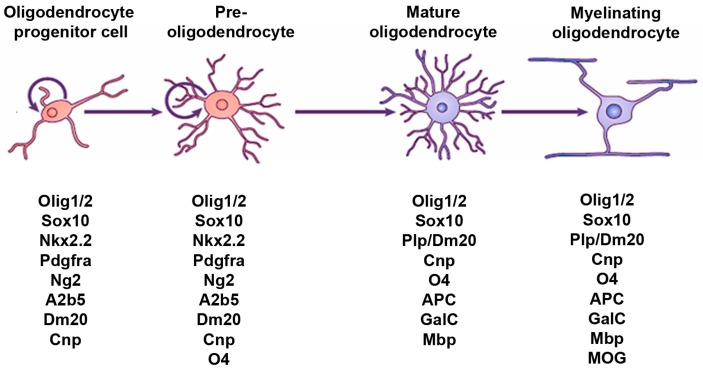
Schematic representation of the developmental stages of the OL lineage. The morphological (top) and antigenic (bottom) features of OPCs, pre-OLs, mature OLs and myelinating OLs are shown. The list of stage-specific markers is restricted to the markers quoted in the present review.

Oligodendrocytes (OLs) are the myelinating cells of the central nervous system (CNS). These cells are consistently found in vertebrates in which they appeared relatively late in evolution in hinge-jawed fishes [1,2]. Contrasting with the wide distribution of OLs in the mature CNS, OL progenitor cells (OPCs) originate at early stages of development in restricted sites bordering the cerebral ventricles and spinal canal before they subsequently migrate into adjacent regions. OPCs express a characteristic set of markers, including the platelet-derived growth factor receptor alpha (Pdgfrα) and the neuron/glial antigen 2 (NG2) proteoglycan. Both markers are rapidly downregulated when the cells differentiate into OLs, unlike other lineage markers as the transcription factors SRY-Box (Sox) 10 and Olig2, which are expressed in both OPCs and OLs. The mature OLs can be identified by their expression of adenomatous polyposis coli (APC) and the myelin basic protein (Mbp), among others (Figure 1). Like neurons and astrocytes, OLs derive from radial glial cells (RGCs), themselves the progeny of the neuroepithelial cells constituting the primitive neuroepithelium [3]. At early stages of neurogenesis, the potential of these primary progenitors becomes regionally restricted through the activity of organizing signals. The latter involve gradients of secreted proteins such as Hedgehog and BMP/Wnt proteins, which emanate from the ventral or dorsal regions of the developing CNS, respectively. The different signals provide positional information to nearby progenitors by upregulating specific sets of transcription factors which will then control cell fate decisions [4].

The generation of the first OPCs takes place in the ventral regions of the CNS and follows the early production of neurons. Additional sources of OPCs subsequently emerge during fetal development in the dorsal CNS and therefore support the existence of different spatiotemporal waves of OL production in the spinal cord as in the brain. The present review describes the involvement of Hedgehog proteins in the development of the OL lineage in the vertebrate spinal cord and brain from the early embryonic stages of development until the neonatal period. While the role of Hedgehog signaling in the ventral oligodendrogenesis has been thoroughly delineated during the last twenty years, the involvement of the pathway in the dorsal oligodendrogenesis was only recently reported in the forebrain and is not a universal situation as shown by the Hedgehog-independent generation of OPCs in the dorsal spinal cord.

## 2. Hedgehog Signaling and the OL Lineage in the Spinal Cord

### 2.1. Hedgehog, a Key Signaling for OPC Specification in the Ventral Spinal Cord

**Figure 2:**
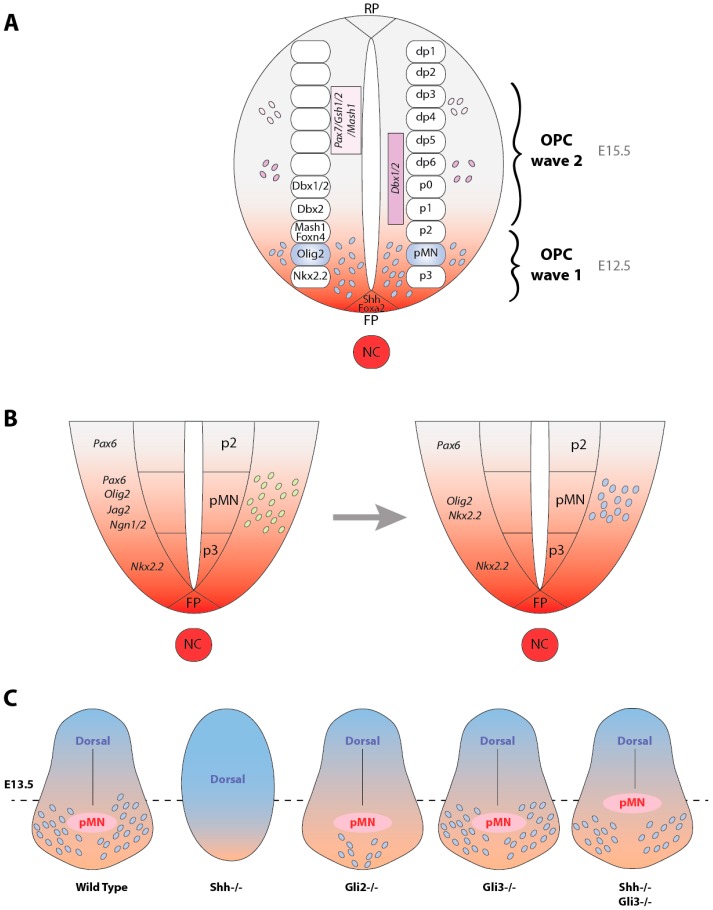
Hedgehog-dependent production of the OL lineage in the developing spinal cord. (**A**) Cross-sectional view of the neural tube indicating the production of the secreted Hedgehog proteins (**red**) from the notochord (NC) and the floorplate (FP). The roofplate (RF) secretes BMP and Wnt. Oligodendrocyte progenitor cells (OPCs) are produced as successive waves, which occur in mice at E12.5 and E15.5, respectively. The first wave arises from the pMN domain and the second one from progenitors expressing either Pax7/Gsh1/2/Mash1 or Dbx1/2. The ventral (p0–p3, pMN) and dorsal (dp1–6) neuroepithelial domains are indicated on the right side of the neural tube while the gene expression patterns for the different ventral progenitor domains are indicated on the left side; (**B**) Schemes of the ventral neural tube during Hedgehog-dependent MN (**left**, in **green**) and OL (**right**, in **blue**) generation from the pMN domain. At the time of OPC specification, Hedgehog signaling maintains Olig2 expression in the pMN domain whereas it promotes the dorsal extension and regression of the Nkx2.2 and Pax6 domains, respectively; (**C**) Schematized views of the neural tube in E13.5 wild-type or knockout mice indicating the distribution of OPCs derived from the pMN. OPCs (**blue**) have already invaded the ventral neural tube in the wild-type mouse. In the absence of Shh, no ventralization of the neural tube is observed and OPCs are completely absent. In the Gli2 mutant, the ventral production of OPCs is highly decreased while it is unaffected in the Gli3 mutant. Finally, the Gli3 mutation partially rescues the ventral oligodendrogenesis in the Shh mutant.

In 1991, Warf and collaborators published the first results suggesting a ventral origin of OLs in the spinal cord [5]. A few years later, separate cultures of the ventral and dorsal regions of the embryonic day (E)14 rodent or E4 chick spinal cord revealed that cells able to generate OLs are restricted to the same domain of the neural tube that gives rise to motor neurons (MNs), the pMN domain [6,7,8,9] (Figure 2A). Based on this observation, two independent groups demonstrated that development of the OL lineage depends on the presence of the notochord (the mesoderm that underlies the ventral spinal cord) and/or the floorplate (the ventral-most spinal cord cell type). Consistently, the Danfourth’s short tail mouse mutant lacking these structures does not display any OPCs at the ventricular surface [10], while chicken embryos possessing a second surgically introduced notochord or floorplate at an ectopic position, show ectopic production of OPCs [10,11]. Since both the notochord and floorplate comprise Sonic hedgehog (Shh)-expressing cells [12], the hypothesis of Shh involvement in the specification of OPCs was proposed. The hypothesis was confirmed by the appearance of OLs in explants of chicken or quail intermediate neural plate (which does not normally give rise to OLs) cultured in the presence of purified recombinant Shh protein. These data were the first ones to strongly support the idea that the specification of the OL lineage depends, at least initially, on ventral inducing signals, including Shh [10,11]. In agreement with this conclusion, the analysis of transgenic mice in which Shh is ectopically expressed in the dorsal midline under the control of the Wnt-1 regulatory element, revealed the induction of numerous Pdgfrα^+^ and O4^+^ oligodendroglial cells in the region adjacent to the source of ectopic Shh [13]. Moreover, the specific Shh antibody mAb5E1 led to detect Shh-expressing cells in the chicken ventral spinal cord immediately prior to and during the appearance of OPCs [14]. The time-window during which Shh is required for the specification of OPCs was evaluated by independent groups by using floorplate ablation and/or blocking Shh antibody. Slightly different results were reported since the period of Shh requirement was alternatively determined as E3–E7.5 [14] and E4.5–E5.5 [15]. The studies also led to suggest that Shh effect is likely direct since at that time, first, the progenitors have lost their competence to generate MNs or floorplate cells in response to Shh and second, the Shh receptor Patched (Ptc) is expressed by the neuroepithelial cells of the ventricular domain producing OPCs [15]. Nevertheless, the mechanisms through which Shh was involved in the emergence of spinal cord OPCs still remained to be investigated.

### 2.2. Hedgehog Transcriptional Targets Leading to OPC Specification in the Ventral Neuroepithelium

The finding that the genes encoding the basic helix-loop-helix transcription (HLH) factors, Olig1 and Olig2, are the first Shh-regulated genes associated with the OL lineage, constituted a substantial progress. Both genes are strongly induced in the ventricular zone (VZ) of mice ectopically expressing Shh in the dorsal neural tube at E14.5, just before the appearance of numerous O4^+^ and Pdgfrα^+^ OL cells in positions adjacent to this domain. The morphogen appeared to be both necessary and sufficient for normal Olig expression. Indeed, the recombinant Shh protein induces a 10-fold upregulation of Olig1 expression when it is added into cultures of neuroepithelial cells derived from E14.5 rat embryos. Moreover, Shh null mice display a complete absence of Olig transcripts in areas classically known to express Shh in the CNS ventral midline [16]. Other investigations led to propose a model according which Shh acts first on multipotential cells to induce Olig1 and Olig2 and, second on committed OPCs co-expressing Olig1, Olig2 and the HLH proteins E2A and HEB. The latter were suggested to facilitate appropriate Olig gene function through a heterodimerization process and to contribute to the control of proliferation and differentiation in the OL lineage. However, the hypothesis still remains to be demonstrated [17].

In a similar manner, Olig gene expression and OL development also require Hedgehog signaling in the zebrafish. In the Smoothened (Smo) mutant (Smu*^b^*^641^) where most Hedgehog signaling is absent, a complete disappearance of trunk expression of Olig2 was first observed in 24 hours post fertilization (hpf) embryos. The mutant also completely lacks spinal cord OLs expressing Plp/dm20 and most MNs consistent with the requirement of Olig2 function for both OL and MN development [18]. The characterization of a second Smo mutant (Smo*^nv^*^122^) similarly revealed the absence of Olig2 expression in the neural tube of 48 hpf-embryos, but also the absence of Olig1 expression which is classically restricted to the OL lineage and starts from 36 hpf while Olig2 is already strongly expressed before 20 hpf [19]. Complete abolition of Olig1, Olig2 and Sox10 expression was also observed in the Dispatched1 mutant Disp*^nv^*^108^ indicating that the loss of long-range Hedgehog signaling mediated by Dispatched blocks OL development [19]. In addition, embryos treated with the Smo inhibitor cyclopamine during various time windows from 6 to 50 hpf exhibit a high to complete absence of Sox10^+^ OLs in the spinal cord. In contrast, when cyclopamine is added from 50 to 62 hpf (after the first Olig1^+^ cells can be detected), no defect in OL formation is observed despite the downregulation of Olig1 indicating that the maintenance of Olig1 expression is Hedgehog-dependant. Remarkably, overexpression of Olig1, Olig2 or both is unable to rescue the lack of OL formation in the absence of Hedgehog signaling, which suggests that the latter has additional critically important roles other than inducing Olig1 and Olig2 expression [18,19].

### 2.3. Do MNs and OLs Arise from a Common Population of Progenitor Cells in Response to Hedgehog Signaling?

The observation that nanomolar concentrations of Shh are as efficient for up-regulating Olig genes or inducing OLs as for inducing MNs from neural tube explants [10,14,16] suggested that both cell types may be derived from a common precursor. Other arguments were also consistent with this hypothesis. First, in the mouse E12.5 embryo, Pdgfrα and Olig-expressing OPCs originate from the ventral most region of the Pax6^+^ domain from which MNs emerge [16]. Then, the analysis of the Olig2^−/−^ mutant mice [20] as well as fate mapping experiments in zebrafish spinal cord [18] showed that Olig2 act as a transcriptional regulator in both MN and OL development.

However, the fact that OLs are generated in spinal cord explants derived from embryos older than those giving rise to MNs and therefore that OLs and MNs are not generated at the same time questioned the previous hypothesis. A first answer to this questioning was provided by the analysis of ventral neuroepithelial explants isolated at various developmental stages of chick embryos. The study showed that the OPC domain comprising O4^+^ and Pdgfrα^+^ cells lies within the most ventral Nkx2.2-expressing domain of the neuroepithelium, and not in the adjacent domain characterized by Pax6 expression from which MNs emerge [15]. Therefore in the chick spinal cord, OL and MN precursors appeared to not share the same transcription factors and consequently to not originate from the same site in the neuroepithelium in contrast to the data obtained in the mouse [16,21]. This was proposed to reflect a species difference in mechanisms of OPC specification in chicken and mouse.

Further investigations in the chicken neuroepithelium partially solved this apparent divergence by showing that the expression of both Olig2 and Nkx2.2 undergoes dynamic changes at the time of OPC specification (Figure 2B). These transcription factors are first expressed in mutually exclusive domains, but then Nkx2.2 expression domain extends in the dorsal direction, resulting in partial overlap of both regions [22,23]. By investigating the mechanisms that initiate and control these changes, Agius and collaborators showed that Shh is sufficient to promote the coexpression of Olig2 and Nkx2.2 in cells of neuroepithelial explants isolated from E5 chick cervico-brachial spinal cord. Shh activity is also necessary for this coexpression since it is required to maintain Olig2 expression in the pMN domain, to promote the dorsal extension of the Nkx2.2 domain and to induce the regression of the Pax6 domain that occurs between E5 and E6. Despite the existence of Shh-mediated proliferation of neuroepithelial cells at these developmental stages, cell proliferation is not the reason for the dorsal extension of the Nkx2.2 domain as shown by the use of the DNA polymerase inhibitor, aphidicolin. Instead, the expansion and regression of Nkx2.2 and Pax6 domains, respectively, result from the repatterning of ventral neuroepithelial cells. In addition to the stimulation of OPC specification, Shh simultaneously restricts the ventral extension of the astrocyte progenitor domain by down regulating the neuroepithelial expression of early markers of the astrocyte lineage [24].

The most recent works, which addressed the question of the uncertain lineage relationship of MNs and OPCs, support the segregating model through which the neuroepithelial cells are intrinsically committed to generate either neurons or glial cells. Wu and collaborators reported a normal number of OPCs after conditional ablation of MN progenitors in the mouse and concluded that MNs and OLs do not share a common lineage-restricted progenitor [25]. Along the same line, in other territories of the mouse CNS such as the diencephalon, the fate mapping of the Plp-expressing cells indicates that the glial cells occurring at E13.5 arise from a new pool of neuroepithelial progenitors distinct from the neuronal progenitor cells [26]. Even more recently, in the zebrafish, the use of a photoconvertible Kaede fluorescent protein expressed by an Olig2 transgene and time-lapse imaging led to the conclusion that the majority of MNs and OPCs arise from distinct progenitor cell lineages and that the MN to OL segregation results from Hedgehog-mediated recruitment of glial-fated progenitors to the pMN domain subsequent to neurogenesis. Concomitantly with MN differentiation, the neuroepithelial cells that originate dorsal to the pMN domain move ventrally and progressively initiate Olig2 expression, characteristic of the pMN identity. Thus, OPCs acquire pMN identity after those that produce MNs. In other words, the ventral sliding of the neuroepithelium brings new cells in range of Hedgehog signals to replenish pMN progenitors that differentiate as MNs [27]. The role of progenitor recruitment from more dorsal populations to maintain the pMN domain remains nevertheless an open question in rodents and birds.

### 2.4. Molecular Mechanisms Associated with the Hedgehog-Dependent MN/OL Segregation

The requirement for Shh to specify OPCs, at a long time after dorsoventral neuronal patterning is completed [14,15,24,28] raised the question of how a single signaling molecule can confer chronologically distinct identities on the same set of ventral neural progenitors. The high increase in O4^+^ cells induced by the artificial rise in the concentration of Shh (25–100 nM compared to 2–25 to classically induce MNs) in the early E1.5 chick neural tube led to propose that the accumulation of the Shh protein at the surface of the ventral neural progenitors is a decisive step in the MN/OL transition [29]. This increase was accompanied by a dose-dependent reduction in the number of MNs differentiating in these cultures suggesting segregation toward an OL fate at the expense of MN production. Similar results were obtained in vivo, after in-ovo electroporation of a Shh-expressing vector at E1.5. In this experimental condition, the Nkx2.2^+^ domain of the VZ was invariably expanded dorsally in the electroporated side. In addition, a premature decrease in expression of the Ngn2 transcription factor involved in neuron differentiation (at E4 instead E5–E6) was observed in the ventral neural progenitors. The detection of a progressive increase in Shh immunoreactivity from E3 to E5 at a level overlaping with Nkx2.2^+^ cells just before OPC induction together with a high level of Ptc expression in this domain were also strong arguments for proposing the involvement of Shh accumulation in the MN/OL segregation [29].

Shh accumulation was attributed to the activity of the sulfatase (Sulf) 1, an enzyme which modulates the sulfation state of heparin sulfate proteoglycans (HSPGs) and starts to accumulate just before OPC specification. In agreement with this hypothesis, Sulf1 overexpression in E4–E4.5 chicken spinal cord dorsally extends the domain of strong Shh staining, highly upregulates Ptc1 transcripts and increases the number of Olig2^+^ cells. On the other side, the addition of Sulf1 blocking antibody at the time of neural plating led to the inhibition of Ptc1 expression usually detected as two bilateral domains in the ventral progenitor zone between E4 and E6. Similarly, OPC specification is severely affected in E12.5 Sulf1-deficient mouse embryos which display abnormal expression profiles for the Shh target genes Ptc1 and Gli1. The absence of Sulf1 expression in Olig2^+^ progenitors as well as the reduction in the number of Olig2^+^ and Sox10^+^ OPCs significantly higher in the double Sulf1^−/−^; Shh^+/−^ mutant than in the single Sulf1^−/−^; Shh^+/+^ mutant led to propose that Sulf1 may act in a non-cell autonomous manner and as a positive regulator of Shh. By eliminating 6O-sulfate groups on heparan sulfate chains, Sulf1 may locally lower Shh/HSPG interaction and promote Shh release from the surface of the progenitors located below the pMN domain. This may increase the amounts of the morphogen subsequently provided to neighboring Nkx2.2^+^ cells before their segregation to an OL fate [29,30]. Sulf1 investigation in the zebrafish spinal cord provided additional data which characterized Sulf1 activity as a timer able to activate a high-threshold response to Hedgehog at the critical time points of neuronal (14 hpf) and OL (36 hpf) generation as indicated by Sulf1 upregulation in the medial and lateral floorplate cells expressing Shh at these time points [31]. Other classes of glycosaminoglycans probably regulate Shh-dependent MN/OL segregation as suggested by the analysis of the keratan sulfate synthesizing enzyme GlcNAc6ST-1 mouse mutant [32].

Shh-mediated regulation of the Notch ligand Jag2 expression is also likely essential to control the timing of the MN to OL segregation. Indeed, Shh restricts Jag2 expression to the pMN domain during the period of MN generation (Figure 2B) as shown by the lack of Jag2 expression in this domain in Shh^−/−^ mice or by the dorsal expansion of Jag2 when a constitutive activation of the Shh pathway is triggered by the use of an activated version of Gli3. Actually, Jag2 activity splits the Olig2 progenitors into two different identities. It prevents a pool of Olig2-expressing progenitors from entering the neuronal differentiation pathway and at the same time, it maintains high expression levels of the Notch effector Hes5 that in turn directly inhibits OPC generation during the neurogenic phase (Rabadan, 2012).

### 2.5. Local Sources of Hedgehog Proteins Contributing to OPC Specification

Although the production of Shh is present in both the notochord and floorplate, the notochord is no longer in contact with the overlying neural tube by E10.5–E11.5. An elegant study led to better evaluate the individual contribution of the floorplate to oligodendrogenesis. The selective inactivation of Shh in the floorplate was performed via mutagenesis approaches using a conditional floxed Shh mouse line and a transgenic strain expressing the Cre recombinase under the Nkx2.2 or Foxa2 cis-regulatory modules (CRMs). In both Nkx2.2^CRM^::Cre;Shh^Flox/Flox^ and Foxa2^CRM^::Cre;Shh^Flox/Flox^ mutants, Shh expression was detected in the notochord but never in the floorplate region of the ventral neural tube. The study of the mutant animals showed that floorplate-derived Shh (Shh^FP^) is required to maintain Shh-Gli target gene and Olig2 expression in the pMN cells between E11.5 and E12.5. As expected by the loss of Olig2 expression at E12.5 in these mutants, the number of Olig2^+^ OPCs is dramatically decreased at E15.5 both in the ventral and dorsal funiculus of the developing spinal cord. During the E10.5–E12.5 time window, the phenotype of these mice is comparable to the phenotype observed in animals exihiting Smo inactivation in Nestin^+^ neural cells indicating that the floorplate is the sole source of Hedgehog proteins required during this period. In contrast, an additional source might be involved in signaling to OPCs destined to migrate to the dorsal spinal cord between E15.5 and E18.5 given the slightly more severe phenotype observed in the dorsal funiculus of the Smo mutant during this time window [33].

In the zebrafish, several Hedgehog proteins other than Shh co-exist in the notochord and floorplate including Indian Hedgehog (Ihhb, previously known as Echidna Hedgehog) in both areas and, Tiggy winckle Hedgehog (Twhh) in the floorplate. The partial compensation of the lost Shh function by Hedgehog homologs throughout the period of MN and OPC specification was proposed accounting for the apparent normal level of Olig2 expression observed in the spinal cord of the syu^−/−^ mutant embryos [18,34,35,36]. In agreement with this hypothesis, in embryos deficient for both Shh and Twhh, spinal cord cells do not express Olig2 and consequently do not produce MNs and OLs as previously observed in embryos deficient for Smo [18,28]. Despite the presence of Olig2^+^ cells in the syu^−/−^ mutant, these cells are nevertheless located more ventrally than normal and do not give rise to OPCs. Thus, Shh and Twhh together appear to induce and position the Olig2^+^ precursor domain. Consistent with these data, the blocking of Hedgehog signaling by a continuous exposition to the Smo antagonist cyclopamine during various time windows including 6–36 hpf (prior to initiation of Olig2 expression), 30–36 hpf (after dorsoventral pattern was established) or 14–26 hpf (during the period of birth of most MNs) also shows that Hedgehog signaling is successively required to induce and position the Olig2^+^ precursor domain, then to maintain Olig2 expression and finally to control the balance between MN and OPC production [28].

More recently, the analysis of Ihhb loss-of-function showed that this protein is also required for OPC specification from the pMN precursors. Indeed, the Ihhb morphant fails to specify Sox10^+^ OPCs at 48 hpf. However, it displays normal Olig2 expression levels suggesting that Ihhb (in contrast to Shh) is dispensable for the maintenance of Olig2 expression. Instead, Ihhb is required for the cell cycle inhibition of the spinal precursors involved in OPC specification as indicated by the increased proliferation of neural precursors and overproduction of neurons at the expense of OPCs during the phase of oligodendrogenesis upon inhibition of Ihhb function. Interestingly, Shh cannot replace Ihhb function in OPC specification confirming that these proteins do have separate functions. The relative expression levels of Ihhb and Shh might be involved in maintaining a precise balance between the proliferation and differentiation of spinal precursors. The underlying mechanism might be that Ihhb behaves as a potent competitive inhibitor of Shh for binding to the Ptc receptor. However, more direct evidence is still needed [34].

Hedgehog expression in the floorplate is a conserved feature in human as well. A high immunoreactive Shh signal can be detected in the floorplate cells at 45 days post coitum (dpc). Shh remains restricted to this region before becoming progressively more weakly expressed at later developmental stages. The first OPCs emerge dorsal to the floorplate at 45 dpc in two discrete regions on each side of the VZ. Later they appear in the ventral and lateral cord, and finally in dorsal regions of the presumptive white matter. Thus, human oligodendrogenesis in the spinal cord apparently displays similarities with chick, rodent or zebrafish oligodendrogenesis [37].

### 2.6. Involvement of the Gli Transcription Factors in OPC Specification

The involvement of the Gli zinc-finger transcription factors in Shh-mediated induction of OPCs was investigated through the analysis of the Gli2 and Gli3 mutants. In the wild-type mouse embryos, Olig1/2 genes are expressed in the pMN domain during neurogenesis (E9.5–E10.5), then downregulated at E11.5 and upregulated again during oligodendrogenesis stages. In the Gli2 mutant, Olig1/2 expression is maintained during the neurogenic step, but its upregulation fails at the oligodendrogenic step (Figure 2C). Therefore, Gli2 activity appears to regulate the size and duration of the Olig1/2^+^ oligodendrogenic domain in the ventral spinal neuroepithelium and the subsequent production of OPCs. Is Gli2 activity directly responsible for the upregulation and maintenance of Olig gene expression during OPC production or does it depend on the supply of Shh from the floorplate? This remains to be determined. Despite a drastic decrease of OPCs at early stages, the steady-state number of these cells in the Gli2 mutant is however quite comparable to that in wild-type littermates at late gestation stages. Gli2 is thus likely not required for the proliferation of OPCs after their initial production. Similarly, Gli2 is probably dispensable for OL differentiation and maturation in the spinal cord since OPC differentiation can be detected in the Gli2 mutant in vivo even though the process is reduced and delayed [38].

The non-essential role of Gli3 in ventral oligodendrogenesis was then suggested by the comparable expression profiles of Olig/Pdgfrα or Pdgfrα/Mbp transcripts dertermined at E13.5 and E17.5, respectively, in the wild-type and Gli3^−/−^ mice [39]. Such a conclusion was quite in agreement with the observation that Gli3 expression becomes restricted to the dorsal spinal cord at E9.5 before the initiation of oligodendrogenesis. However, the Gli3 null mutation was interestingly found to induce a substantial rescue of the defect observed in the Shh^−/−^ mice or in the mutant diplaying a specific removal of Shh in the floorplate. Therefore, Shh is proposed to maintain Olig2 expression in OPCs via the repression of the antagonistic Gli3 repressor activity [33,39]. In contrast, Nkx2.2 expression being not influenced by the presence or the absence of Gli3 in embryos lacking Shh in the floorplate, Shh^FP^ is likely primarily required to induce Nkx2.2 expression in OPCs via the Gli activators rather than via inhibition of the Gli3 repressor [33]. Finally, the suppression of OL differentiation reflected by the absence of Mbp^+^ Plp^+^ OLs in the E18.5 double Shh^−/−^Gli3^−/−^ mutant supports the involvement of components other than Gli3 in the last steps of OPC maturation [39,40].

### 2.7. Sources of OPCs Outside the pMN Domain Are Hedgehog-Independent

The exchange of isotopically and isochronically defined sectors of the E2 spinal cord between quail and chick embryos led to the first description of a pool of OPCs arising in the dorsal spinal cord [41]. This finding was consistent with the generation of OPCs observed in vitro upon exposure of the dorsal neural tube to notochord explants or to Shh [8,14]. The existence of a second wave of OLs (Figure 2A) was then independently reported by several groups. The data supporting this hypothesis were based on the detailed expression profiles of the Plp/dm20 marker [42,43], the existence of FGF2-responsive stem cells able to generate OLs [44], the description of a subpopulation of Dbx-derived OPCs three days later than the majority of OPCs generated from the pMN [45] and the report of OPCs arising at E15.5 and differing from their ventral counterparts in the requirement for Nkx6. These latter progenitors co-express Pax7, Gsh1/2, Mash1 and were proposed to potentially result from a dorsal evasion of BMP signaling [46,47], in agreement with the tightly regulated balance between ventral Shh and dorsal BMP activities, which influence the position at which OPCs are specified in the neural tube [48,49]. The Shh-dependent induction of Olig1/2 expression in the pMN domain first raised the possibility that Shh also mediates the induction of Olig1/2 expression in the dorsal neural tube. However, OL generation from the dorsal domains appears to be independent of Shh signaling. First, the Smo antagonists, cyclopamine or KAAD-cyclopamine, do not prevent the differentiation of the FGF2-responsive stem cells into O4^+^ Galc^+^ OLs. In the same line, Olig2^+^/Nkx2.2^+^ OPCs are induced in the presence of FGF2 in primary cultures of embryonic spinal cords derived from Shh^−/−^ mice [44]. In this mutant where most of the ventral structures including the pMN are missing, no early OPCs are produced at or before E13.5. However, a small number of Olig1/2^+^ cells start to appear in the dorsal spinal cord at E14.5. A larger number is observed by E18.5 with nevertheless a delay in OL lineage progression as indicated by the late appearance of Pdgfrα and Sox10 expression at this developmental stage and by the complete absence of Mbp expression at perinatal stages [46]. Finally, dissociated spinal cord cells derived from E12.5 embryos which express an inducible GFP reporter under the Dbx1 promoter give rise to GFP^+^Sox10^+^ OL cells in the presence of cyclopamine, but not in the presence of an inhibitor of the FGF receptor [45]. Recently, the generation of a dual reporter mouse line to color code ventrally and dorsally-derived OPCs and their differentiated OL progeny led to show that 80% of OL lineage cells in the postnatal spinal cord are ventrally-derived in a Hedgehog-dependent manner. Ventral OPCs appear early and spread uniformly throughout the cord, whereas dorsal OPCs arrive later and remain mainly in the dorsal and dorsolateral funiculi. Remarkably, ventrally and dorsally-derived OPCs/OLs do not display distinct electrical properties [50].

## 3. Hedgehog Signaling and the OL Lineage in the Brain

**Figure 3:**
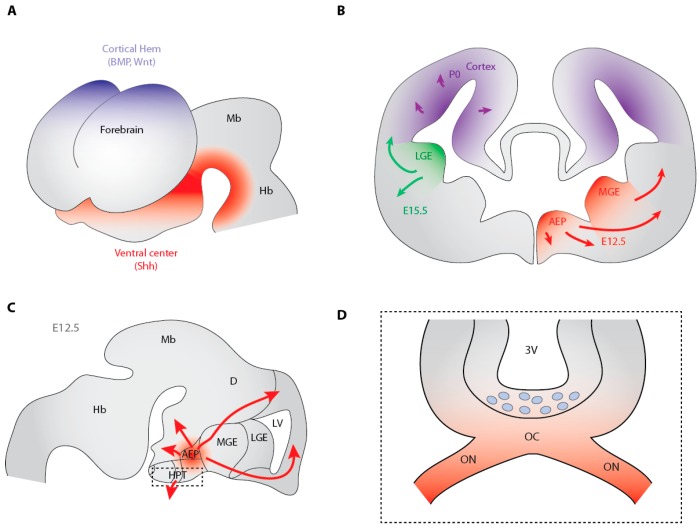
Hedgehog-dependent generation of the OL lineage cells in the brain. (**A**) Scheme visualizing the ventral (**red**) and dorsal (**blue**) organizing centers of the forebrain which secrete Hedgehog and BMP/Wnt, respectively; (**B**) Cross-sectional view of the mouse telencephalon indicating the regions of the ventricular zone where the different waves of OPCs are generated. The earliest wave (E12.5, red) arises from Nkx2.1-expressing precursors, which appear in the anterior entopeduncular area (AEP) and medial ganglionic eminence (MGE) and then migrate into all parts of the telencephalon. The second wave derives from Gsh2^+^ precursors located in the lateral ganglionic eminence (LGE, green) at E15.5. The last wave emerges from Emx1-expressing cortical precursors (**blue**) around birth; (**C**) Sagittal view of the brain indicating the position of the developing hypothalamus which also generates OPCs in E12.5 mouse embryo; (**D**) Coronal view of the hypothalamic region indicated by the boxed area in (**C**). The localization of the Shh protein (red) transported through the optic nerves (ON) towards the optic chiasm (oc) is shown. The OPCs (**blue**) generated in the floor of the third ventricle (3V) in a Hedgehog-dependent manner at E12.5 in mice are aimed at colonizing the optic nerves. D, diencephalon; Hb, hindbrain; LV, lateral ventricle; Mb, midbrain.

The origin of OLs at more anterior levels of the neuraxis has long remained less well established than in the spinal cord. Plp/dm20^+^ cells were first described in the ventral neuroepithelium of the embryonic mouse diencephalon at E9 [51]. Then, a cluster of Pdgfrα^+^ presumptive OPCs able to proliferate and migrate was reported at E14 in the ventral forebrain of the rat embryo [9]. Like the developing spinal cord, the developing brain exhibits signaling centers, including the ventral center and cortical hem which constitute a source of Hedgehog and BMP/Wnt proteins, respectively (Figure 3A). Therefore, as previously done in the spinal cord, the implication of Hedgehog signaling in OPC generation was progressively investigated at the different caudo-rostral levels of the developing brain.

### 3.1. Hedgehog-Dependent Generation of OPCs in the Hindbrain and Midbrain

As previously observed in the spinal cord, the ventral VZ is the site where the first OPCs are detected in the chick metencephalon, the embryonic part of the hindbrain that differentiates into the pons and the cerebellum [52,53]. In this region, O4^+^ cells are present rostrally in bilateral ventricular foci adjacent to the ventral midline at stage 26 (E5). Subsequently, OPCs populate lateral and dorsal regions in a rostral to caudal manner. At stage 30 (E6), these cells reach rostrally the area of the presumptive lateral pons whereas they dispersed caudally at the transitional area between the lateral pons and the cerebellar anlagen. OPCs are then detected in the dorsal parenchyma (cerebellar anlagen) by stage 32 (E7.5) and in the entire metencephalon by stage 34 (E8). Thus, the avian metencephalon is characterized by a rostro-caudal and a ventro-dorsal pattern of OPC appearance [52]. The ventral VZ is not the unique source of OPCs detected in this brain region, since additional discrete ventricular domains are detected in more lateral and dorsal locations that subsequently develop O4^+^ cells. In those domains, OPCs appear several stages later after those in the ventral VZ and in vitro studies suggest that approximately 20% of OPCs are induced during these later stages [52].

Interestingly, all these OPC domains are correlated with the transient expression of Shh in adjacent tissue suggesting that Shh signaling may regulate the spatial restriction of OPC appearance. In agreement with this hypothesis, Shh signaling is first required for the proper development of the earliest OPCs in the chick metencephalon since the injection of mAb5E1-producing hybridomas at stage 19 led to a dramatic decrease of OPCs throughout the tissue. In contrast, exposure to the neutralizing antibody at stage 23 inhibited the subsequent appearance of O4^+^ cells in the lateral and dorsal VZ but not in the ventral midline suggesting that the former zones contribute to the dorsal pool of OPCs. Dissociated cells derived from the metencephalon of embryos at stage 20, 24 or 26 and then cultured in the presence of mAb5E1 led to a reduction of O4^+^ cells by 92, 80 and 19%, respectively, indicating that the initial appearance and survival of most OPCs become independent of Shh signaling between stage 24 and 26 in vitro. In purified cultures of OPCs, Shh promotes cell survival and proliferation, which suggests that the morphogen can act directly on these cells. Finally, the spatial restriction of OPC appearance can be disrupted by the addition of exogenous Shh [52].

Similarly, in the rodent hindbrain, OLs are first generated from ventral progenitor domains while a subset of OPCs also derives from the dorsal hindbrain in a region located immediately dorsal to the Dbx2 expression domain co-expressing Pax3, Pax7 and Gsh1 [47]. The specification of ventral hindbrain OPCs was more accurately analyzed and showed to have a similarity with the ventral spinal cord OPCs with respect to their dependence on Shh signaling and requirement for Olig1/2, the expression of which extends until the second rhombomere of the hindbrain [16,54]. However, Vallstedt and collaborators revealed crucial differences in the intrinsic programs that control Olig1/2 expression in the rodent ventral spinal cord and anterior hindbrain. Indeed, Nkx6 proteins mediate opposing effects on the generation of OPCs in both regions since they are required for OPC generation in the ventral spinal cord while they instead suppress OPC production in the ventral anterior hindbrain. This differential regulation appeared to be tightly linked to the expression of Nkx2.2. Consistent with this hypothesis, Olig2^+^ cells are generated dorsal to the Nkx2.2 domain in the spinal cord whereas in the anterior hindbrain where they are first detected at E12.5, these cells co-express Nkx2.2 indicating that activation of Olig1/2 expression at different positions of the developing neural tube is regulated by distinct genetic programs [47].

In the zebrafish, the homozygous Smo mutant completely lacks hindbrain OLs expressing Plp/dm20 [18]. In addition to the expression of Olig2, Nkx2.2a expression also depends on Hedgehog signaling in the zebrafish hindbrain as shown by the complete absence of Nkx2.2a transcripts in this region in the same mutant. Interestingly, the homozygous histone deacetylase 1 (Hdac1) mutant also displays a complete absence of Olig2^+^ and Sox10^+^ cells at 50 hpf, but on the contrary an upregulation of Nkx2.2a expression. As the double Smo;Hdac1 mutant also fails to express Nkx2.2a in the ventral hindbrain, Nkx2.2a expression seems to require Hedgehog signaling irrespective of whether embryos are deficient in Hdac1 function or not. Thus, Hdac1 likely facilitates Hedgehog-mediated expression of Olig2 in the ventral hindbrain and represses the expression of Nkx2.2a in the ventral neural progenitors en route to the production of Sox10^+^ OPCs [55]. An additional unexpected target of Shh signaling during OPC specification in the zebrafish hindbrain is Disrupted-in-schizophrenia1 (Disc1). In agreement with this observation, cyclopamine blocks Disc1 expression in Olig2^+^ midline progenitor cells and also mimics the effect of Disc1 knockdown on OPC specification. Interestingly, these data led to suggest for the first time that altered Shh signaling may be a potential important developmental factor in the pathobiology of mental illnesses [56].

The development of OPCs in the chicken midbrain or mesencephalon has also been examined. It starts ventrally at around E6 when some Olig2^+^ cells emerge from the Nkx2.2^+^ neuroepithelial cells in ventrolateral positions. At around E11, Olig2^+^ OPCs then emerge from the VZ and subventricular zone (SVZ) of the dorsal midbrain. Unlike Olig2 staining, Nkx2.2 expression is not detected in the VZ/SVZ, but in Olig2^+^ cells migrating away from their origin. These Olig2^+^ Nkx2.2^+^ cells coexpress the OL markers Pdgfrα and Sox10 and a small number continues to cycle during the migration process. In both the ventral and dorsal VZ of the midbrain, Shh is expressed in the vicinity of Olig2^+^ OPCs consistent with the possible Shh involvement in the generation of these cells [57]. Moreover, a small region of the mesencephalic neuroepithelium called the parabasal band, was recently proposed to be the source of most OPCs present in the cerebellum [58]. Hedgehog implication in the specification of this main population of OPCs remains to be precisely addressed. However, insight on the molecular mechanisms underlying OL development in this brain area was provided by a recent study showing that during early postnatal development, Shh stimulates the proliferation of Olig2^+^ OPCs and downregulates their differentiation. Thus, Shh may prevent cerebellum OPC from exiting the cell cycle and inhibit the effects of factors promoting their differentiation. In agreement with this hypothesis, by the end of the first postnatal week, Purkinje cells downregulate Shh and produce vitronectin which induces OL maturation [59].

### 3.2. Hedgehog-Dependent Production of OPCs in the Ventral Telencephalon

At the end of the 1990s, several publications started to investigate OPC genesis in the forebrain. Precursor cells derived from E15 rat striatum either cultured in vitro or transplanted into the eye were shown to have a greater capacity to generate OPCs than precursors from the neocortex [60,61]. Moreover, histological evidence for a ventral source of OLs in the rodent and chick forebrain was reported by establishing the expression profiles of the Plp/dm20 and Pdgfrα OPC markers, respectively [42,62]. Since a region of the ventral forebrain was appearing to be specialized for the generation of OPCs, several groups were incited to further define the location of this region and started to explore more accurately its establishment. The data which are presented below support the idea that in the ventral telencephalon, as previously shown in the developing ventral spinal cord and caudal brain, Shh is likely one of the factors which are important for the generation of OPCs with nevertheless a higher level of complexity [54,63,64,65].

The first argument was the localization of the Pdgfrα expression domain within a region of the anterior hypothalamic neuroepithelium that co-express Shh and Ptc from E13.5 to E17 in the rat. These expression profiles suggested that Pdgfrα^+^ progenitors, before spreading to the forebrain into areas where Shh is not expressed (such as the cerebral cortex), are exposed and respond to Shh. Loss- and gain-of-function experiments supported this hypothesis. The Nkx2.1 null mutant mice which lack the telencephalic Shh expression domain display no Pdgfrα^+^ OPCs in the forebrain VZ. In contrast, early OL markers are not lost in areas of the Nkx2.1 mutant forebrain where Shh persists such as the zona limitans intrathalamica and the amygdaloid region [63]. Consistent data were obtained in the zebrafish with a lower level of Olig2 expression in the brain of the syu^−/−^ mutant embryos, which are deficient for Shh [18]. In vivo gain-of-function experiments using the ectopic expression of Shh in the telencephalic neuroepithelium of E9.5 mouse embryos led to the production of a majority of OLs at the expense of neurons. Interestingly, in P21 postnatal mice, Shh-infected cells express myelin markers in the white matter but rarely in the grey matter, suggesting that while Shh can direct cells to an OL fate, local cues are involved in allowing these cells to adopt a mature OL phenotype. In addition, in favor of the role of Shh in OPC production in the ventral telencephalon was the observation that in vivo Shh gain-of-function can partially rescue the failure of OL development in the Nkx2.1 mutants and notably induce Olig2 and Pdgfrα^+^ expression ectopically in the dorsal cortical regions in the mutant as in the wild-type animals [63].

Complementary culture systems revealed nevertheless a higher level of complexity for the Shh-dependent generation of OPCs in the forebrain. As expected, the Smo antagonist cyclopamine inhibits OPC development in cultures of mouse ventral telencephalon. However, OPCs were found to develop in cultures of Nkx2.1^−/−^ basal forebrain which lack Shh expression. Even more surprising was the ability of cyclopamine to block OPC generation in those cultures. Similar results were obtained when different regions of the telencephalon including the median ganglionic eminence (MGE), the lateral ganglionic eminence (LGE) and the cortex were cultured as either explants (from E13.5 embryos) or dissociated cells (from E17.5 embryos). All regions were qualitatively similar in their ability to generate OPCs regardless the embryo genotype thus suggesting that Shh is apparently not required for OPC production in vitro [63,65]. Several hypotheses were proposed to account for these results including the presence of Shh and Ihh in the cultures. However, the reasons for Shh-independent generation of OPCs in vitro remained nothing less than surprising.

### 3.3. Cooperation between Shh and Fibroblast Growth Factors in the Generation of Ventral Telencephalon OPCs

The revisiting of Shh-dependency of forebrain OPC production allowed the identification of a population of Pdgf-responding precursors (PRPs) isolated from E14 mouse MGE and able to respond to Shh signaling. In concert with Pdgf signaling, Shh induces proliferation and/or survival of these cells. In addition, these precursors are able to generate neurospheres which give rise both to neurons and OPCs suggesting that a common precursor can generate these two cell types in the ventral forebrain. The self-renewal of these precursors is cooperatively increased in the presence of both Pdgf and Fgf2. This effect being reduced by the presence of cyclopamine, the self-renewal of PRPs was finally proposed to depend on the effective activation of Shh signaling by both Pdgf and Fgf2 [66]. In the same line, Shh and Fgf2 induce E13.5 mouse neocortical precursors to express the transcription factor Olig2 and to generate OPCs in culture. These shared activities for inducing OPCs involve overlapping intracellular signals. Indeed, the induction of Olig2 by Shh or Fgf2 is strongly inhibited by specific inhibitors of MEK/MAPK or PI3K pathways indicating that these pathways are crucial for the first step of OPC specification. However, only Fgf2 is able to increase MAPK activity. Since Shh effect is strongly inhibited by both cyclopamine and a specific inhibitor of the FGF receptor tyrosine kinase, Shh effect likely depends on Fgf activity for maintaining a basal level of phosphorylated MAPK [67]. The conditional deletion of Fgf receptors (Fgfr) 1 and 2 in the mouse embryonic forebrain then revealed that in vivo Fgf signaling through the cooperation between both receptors is required for the initial generation of OPCs in the ventral forebrain. Since the failure of OPC generation in the Fgfr mutants occurs without loss of Shh signaling and the pharmacological inhibition of either Fgfr or Hedgehog signaling in parallel cultures strongly inhibits OPC generation, it was concluded that Fgfrs cooperate with Shh to generate OPCs [68]. Crosstalk between Shh and Fgf signaling pathways is not restricted to the rodent forebrain, since in the zebrafish, Fgf16 which is required for OL development notably in the forebrain, is induced by the Hedgehog signaling [69]. However, a notable difference might exist in human since FGF2 was there proposed to inhibit the transition of pre-OPCs to OPCs by repressing SHH-dependent co-expression of OLIG2 and NKX2.2 [70].

### 3.4. OPC Production is Gli2-Independent in the Forebrain

The role of the Gli2 transcription factor in Shh-mediated induction of OPCs was investigated in the mouse forebrain. In contrast to the spinal cord, OL development in the forebrain remains unaffected by Gli2 mutation despite Gli2 expression in this brain area [38]. At E12.5, Shh expression is mainly detected in the mantle zone of the MGE, anterior entopeduncular area (AEP) and hypothalamus both in the wild-type (Figure 3B,C) and the Gli2 mutant embryos with nevertheless a slight difference in the hypothalamus where Shh expression is restricted to the midline in the mutant likely due to the loss of some ventral tissue. No obvious difference is detected in the number or distribution pattern of Olig2^+^ and Pdgfrα^+^ OPCs. The latter are highly detected in the VZ of MGE and to a lesser extent in the LGE. A few migratory OPCs are also observed in the mantle of the MGE. At E14.5, numerous Olig2^+^ and Pdgfrα^+^ OPCs are found in the striatal and septal regions in both phenotypes with some OPCs already spreading dorsally into the neocortex. If Gli2 activity thus appears to be dispensable for Shh expression in the ventral telencephalon and OPC specification in the forebrain, it remains nevertheless unclear whether Gli2 can play a role in the terminal differentiation of OLs. The apparent unaltered generation of OPCs in the Gli2 mutant forebrain was proposed to be consistent with the possible redundant functions of Gli1 and Gli2 in regulating Shh expression [71] and thus oligodendrogenesis in the forebrain [38].

### 3.5. The Dorsally-Derived Third Wave of OPC Production in the Telencephalon Depends on Shh

Numerous questions remained unanswered in the forebrain such as the existence of a single homogenous population of OPCs or the involvment of Shh in the generation of all OLs. The finding that competitive waves of OLs exist in the forebrain corresponded to an important progress in our understanding of OPC production in this brain area. By using a Cre-lox fate mapping approach in transgenic mice expressing the Cre recombinase under the control of Nkx2.1, Gsh2 and Emx1, Kessaris and collaborators showed that the first OPCs originate in the AEP and MGE in the ventral forebrain at E12.5. From these regions, they populate the entire embryonic telencephalon including the cerebral cortex just before the appearance of a second wave of OPCs from the LGE at E15.5. A last wave finally arises within the perinatal cortex (Figure 3B). The destruction of one of these populations surprisingly induces the remaining cells to take over and compensate for the lost population [72]. Consistent with the much expanded cortex in the mammalian, the dorsal wave of OPC production exists in rodents but not in birds where all OLs in the cortex were found to arise from ventrally-derived, migratory OPCs [73]. The dual reporter mouse line previously described in the context of the spinal cord and which allowed tracing ventrally and dorsally-derived OPCs and their progeny, led to show that only 20% of OLs in the postnatal corpus callosum are ventrally-derived. This pattern is thus different from the observation done in the developing spinal cord, where most OPCs arise from the ventral VZ under the influence of Hedgehog signaling, whereas a minority is generated from the dorsal VZ in a Hedgehog-independent manner [50].

The hypothesis that ventral OPC appearance depends on Hedgehog signaling while dorsal OPC do not (as previously proposed in the spinal cord but possibly not in more caudal regions of the brain) was questioned by several publications in the telencephalon. For instance, Smo removal in the neural progenitors which express the ubiquitous marker Nestin as soon as E12.5, leads to OL deficiency in the early postnatal telencephalon suggesting Smo involvement in the generation of pallial-derived OLs [74]. Moreover, in vitro data reported that neonatal purified cortical OPC cultures are able to respond to Shh which induces a dose-dependent increase of thymidine incorporation in these progenitors [75]. More recent data now provide the demonstration that a dorsal Shh-dependent domain in the SVZ produces large numbers of OLs in the neonatal brain [76]. This domain borders the developing corpus callosum and transiently expresses the Shh target gene Gli1 and the transcription factor Pax6. The use of adenoviral lineage tracing to label dorsal RGCs expressing Gli1 in neonates led to detect 28 days later, fluorescent cells expressing Olig2, Sox10 and APC (a marker of mature OLs). In addition, the genetic ablation of Smo or in contrast, the expression of the constitutively active Smo M2 receptor in dorsal RGCs resulted in a high reduction or increase, respectively, in the production of OPCs/OLs in the corpus callosum [76]. In parallel and in an unexpected manner, the Gli1 null mice were reported to start myelination in the corpus callosum earlier than the wild-type animals suggesting that Gli1 delays the onset of myelination [77]. This apparent discrepancy will obviously have to be clarified. Interestingly, in adulthood, the ectopic activation of Smo in the dorsal SVZ or the delivery of Shh into the lateral ventricle still promotes oligodendrogenesis in the corpus callosum. In that case, the Shh protein detected in the SVZ is likely produced by various areas of the ventral forebrain and transported through projecting axons or the cerebrospinal fluid [76,78].

In human at midgestation, OPCs originate both in the ventral telencephalon and in the cortical SVZ [79,80]. The OL lineage cells can be detected by markers similar to those used in other vertebrates. The human fetal RGCs isolated from the cortical SVZ are also able to generate OLs in vitro and the production of these cells is enhanced by Shh [79,81,82]. However, the inhibition of endogenous Shh signaling with the Smo antagonist cyclopamine does not reduce the density of Olig2^+^ cells suggesting the existence of an additional Shh-independent mechanism for human OL generation at least in vitro [82].

### 3.6. Antagonistic Activities of Shh and BMP on the Transcriptome of Telencephalon OPCs

A tightly regulated balance between Shh and BMP has been reported for OPC differentiation in the spinal cord where BMP represses Shh-target genes Olig2 and Nkx2.2 [48]. In a similar manner, investigation of the effects of those morphogens was performed on A2B5^+^ OPCs prepared from rat neonatal cortices. In the presence of Shh and BMP4, A2B5^+^ OPCs give rise to O4^+^ late OPCs and GFAP^+^ astrocytes, respectively. These effects are associated with morphological changes including the appearance of multiple thin processes and rounding up of the soma for Shh-treated precursors and, few thick cytoplasmic expansions together with an enlargement and flattening of the cell body for BMP4-treated cells. Shh also induces the occurrence of electron-dense aggregates distributed along the nuclear periphery whereas BMP4-treated cells retain a dispersed and homogenously distributed euchromatin. These opposite effects on chromatin compaction were proposed to be likely mediated by a differential regulation of histone acetylation. Hdac1 and 2 appear to repress astrocytic genes during Shh-induced OL differentiation, but to be dispensable for BMP4-induced astrogliogenesis. In OPCs, Hdac activity would thus be responsible for the repression of other lineage genes. Moreover, an interesting heat map representation of gene expression profiles revealed that the progression from OPC to more mature OLs requires fewer transcriptional changes than the conversion to astrocytes since 1200 and 14000 genes are regulated by Shh and BMP4, respectively [83].

### 3.7. Shh-Dependent Generation of OPCs in the Optic Nerve

In the optic nerve, OPCs are derived from cells located in the diencephalon, especially in the floor of the third ventricle directly dorsal to the optic chiasm (Figure 3C,D). These cells or their progeny populate the optic nerve before becoming differentiating and myelinating OLs [53,84]. The mechanism of the specification of optic nerve OPCs remained unclear until 2006 when Gao and collaborators demonstrated that the appearance of chick OPCs is temporally correlated with and dependent on retinal axon projection to the brain in vitro and in vivo [85]. The observation that the arrival of retinal axons to the forebrain occurs at stages 27–29 (E5–E6) concomitantly with the initial appearance of OPCs incited to perform co-cultures of dorsal spinal cord and retina explants. These cultures indicated that signals derived from the developing retina are competent to induce OPCs in a responsive tissue. One of the molecular cues expressed by retinal axons is Shh. Its expression is correlated spatially and temporally with the axonal growth of the optic nerve towards the optic chiasm. Shh is synthesized in the retinal ganglion cells which are the source of the protein which is then transported via the retinal axons. The Ptc receptor is also strongly expressed in the floor of the third ventricle at the time of OPC specification. These expression profiles as well as the complete inhibition of the OPC inductive activity of retinal conditioned medium by either cyclopamine or the specific Shh antibody mAb5E1 led to propose that the axonal induction of OPCs depends on Shh. In a consistent manner, in vivo injection of cyclopamine at embryonic stage 19 (E2) and analysis at stage 31 (E7) when O4^+^ cells are clearly detectable at the ventral midline of the third ventricle, revealed a dramatic reduction in these cells. The treatment of explants isolated at stage 27 (E5) also led to a decrease in OPCs indicating that OPC induction depends on continued Shh signaling. However, although Shh is likely required for the appearance of optic nerve OPCs, it is probably not sufficient for the commitment of neural epithelial cells to the OL lineage [85].

The distribution of Shh signaling components at later stages of development notably when OPCs colonize the optic nerve suggested that the pathway may contribute to other aspects of OPC development such as their proliferation or migration. In the mouse, scattered precursors are first detected in the proximal end of the nerve at E14.5. They reach the distal end at E16.5 and are homogenously distributed along the entire nerve by E17.5 [86]. During this period, Shh is still expressed by the retinal ganglion cells and transported along their axons [87,88,89]. The ventral portion of the third ventricle of E14.5 mouse embryos expresses high levels of Gli1 transcripts which are maintained in the committed OPCs therefore still competent to respond to Shh signaling. The colonization of the optic nerve by OPCs occurs when the majority of the retinal ganglion cell axons have already reached the forebrain. This process is under the control of several growth factors and axon guidance molecules notably promoting OPC mobility along the optic nerve, while BMPs prevent OPCs from invading the neural retina [90]. Cultures of optic nerve explants from E16.5 mouse embryos led to demonstrate that Shh induces the proliferation of optic nerve OPCs and acts as a chemoattractant for their migration. OPC migration is blocked by the specific mAb5E1 Shh antibody. Once they have colonised the optic nerve, OPCs lose their chemoattractive but not their proliferative response to Shh. Shh-dependency of OPC migration was also shown in E4.5 chick embryo by *in ovo* interference with Shh signaling. How newly generated OPCs are not retained in the floor of the third ventricle where Shh protein is also present remains however an open question [91].

The possible involvement of other components of Shh signaling was then addressed notably by the investigation of the role of the multiligand receptor megalin, a member of the low-density lipoprotein receptor family. An interesting observation is that the expression of megalin, Ptc and Gli1 seems to parallel the OPC colonization of the optic nerve from the chiasm to the retina. In the optic nerve, megalin is exclusively expressed in astrocytes at the time when OPCs colonize this structure, whereas Ptc and Gli1 are found in Olig2^+^ cells. The capacity of megalin neutralizing antibodies to block the effects of Shh on the migration and proliferation of optic nerve OPCs suggests that megalin takes part in these effects. The proposed mechanism is that Shh is internalized via the multiligand receptor before being released by astrocytes to promote the migration and proliferation of OPCs. Megalin might thus control the level of Shh available for OPCs along a defined gradient during the colonization of the optic nerve [92].

## 4. Conclusions

The data accumulated over the years on the role of Hedgehog signaling in the genesis of the OL lineage clearly demonstrates that the pathway, among others, occupies an important position in this developmental process. Hedgehog signaling involvement in OPC production is conserved across vertebrates including human and regards the whole CNS. Despite that, several questions remain notably in the brain where OPC production exhibits a level of complexity higher than in the spinal cord due to the diversity of the cerebral structures. Interestingly, whatever the region of the CNS which is considered, the ventrally (spinal cord) or dorsally (forebrain)-derived OL population that predominates appears to be generated in a Hedgehog-dependent manner. Therefore, a better understanding of the crosstalks existing between Hedgehog signaling and other signaling pathways as well as the identification of the Hedgehog pathway components which transduce Hedgehog signals should improve our knowledge of the molecular mechanisms which are involved in OPC development and ultimately open new perspectives in myelin diseases.
